# Bi-metallic electrochemical deposition on 3D pyrolytic carbon architectures for potential application in hydrogen evolution reaction

**DOI:** 10.1080/14686996.2024.2421740

**Published:** 2024-10-29

**Authors:** Prince Kumar Rai, Amritanshu Singh, Shashwat Bishwanathan, Prashant Kumar Gupta, De-Yi Wang, Monsur Islam, Ankur Gupta

**Affiliations:** a Department of Mechanical Engineering, Indian Institute of Technology, Jodhpur, India; b Department of Chemical Engineering, Indian Institute of Technology, Jodhpur, India; c IMDEA Materials Institute, Getafe, Madrid, Spain

**Keywords:** Pyrolytic carbon, architected material, electrochemical deposition, 3D printing, conducting architectures

## Abstract

3D printing has emerged as a highly efficient process for fabricating electrodes in hydrogen evolution through water splitting, whereas metals are the most popular choice of materials in hydrogen evolution reactions (HER) due to their catalytic activity. However, current 3D printing solutions face challenges, including high cost, low surface area, and sub-optimal performance. In this work, we introduce metal-deposited 3D printed pyrolytic carbon (PyC) as a facile and cost-effective HER electrode. We adopt an integrated approach of resin 3D printing, pyrolysis, and electrochemical metal deposition. 3D printing of a resin and its subsequent pyrolysis led to 3D complex architectures of the conductive substrate, facilitating the electrochemical metal deposition and leading to layered 3D metal architecture. Both monolayers of metals (such as copper and nickel) and bi-metallic 3D PyC structures are demonstrated. Each metal layer thickness ranges from 6 to10 µm. The metal coatings, particularly the bi-metallic configurations, result in achieving significantly higher mechanical properties under compressive loading and improved electrical properties due to the synergistic contributions from each metal counterpart. The metalized PyC structures are further demonstrated for HER catalysts, contributing to the development of highly efficient and durable catalyst systems for hydrogen production. Among the materials studied here, Ni@Cu bimetallic 3D PyC electrodes are particularly well-suited, demonstrating a low HER overpotential value of 264 mV (100 mA/cm^2^, KOH (1 M)) with corresponding Tafel slopes of 107 mV/dec, with exceptional stability during a 10 h operation at a high applied current of −50 mA/cm^2^.

## IMPACT STATEMENT

This study introduces electrodeposited bilayer copper-nickel coated 3D printed pyrolytic carbon structures as cost-effective electrodes and demonstrates their performance as effective electrocatalysts in hydrogen evolution reaction applications.

## GRAPHICAL ABSTRACT

**Figure:**
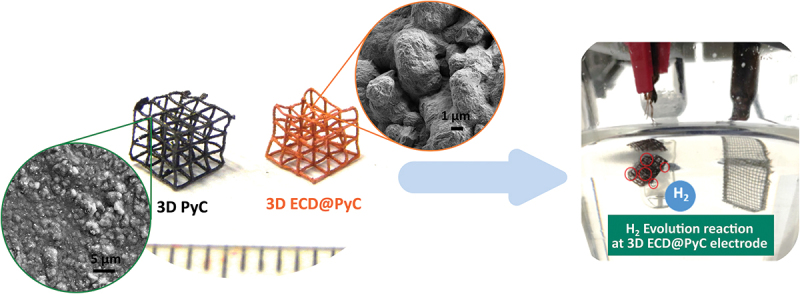

## 1. Introduction

Climate change and the energy crisis are pressing global worries that need immediate solutions in the form of reducing carbon emissions and carbon neutrality. Adopting renewable energies and a sustainable economy are two of the emerging approaches toward reaching these solutions. Hydrogen produced by water electrolysis is a highly appealing pathway for producing clean energy due to its environmentally favorable characteristics, high calorific value, and maximum gravimetric energy density (142 MJ/kg at 25°C) [1]. In this method, hydrogen evolution reaction (HER) and oxygen evolution reaction (OER) occur on the cathode and anode surfaces, respectively [2]. The electrodes for achieving appropriate electrocatalytic HER activity should feature properties including large active surface area, electrochemical stability, good electrical conductivity, low overpotential, and low-cost properties [2–4]. The current benchmark for HER electrodes is platinum (Pt). However, it has significant limitations in practical uses due to its high cost and limited durability [5]. Several transition metals have recently been explored as promising low-cost alternatives. Among them, nickel (Ni) is one of the most popular choices for HER cathodes in water electrolysis, owing to its outstanding catalytic activity, robust stability in alkaline conditions, and cost-effectiveness. Furthermore, compared to Ni electrode, a binary or ternary hierarchical alloy based on Ni exhibits improved catalytic activity toward HER [2,6–8]. Of interest, alloying with copper (Cu) has been a popular choice due to the easy processability and high electrical and thermal stability of Cu. Additionally, a porous Ni – Cu coating boosts the electroactive surface area, further enhancing HER electrocatalytic performance [2,9,10]. The 3D structures, with their high surface areas, also contribute significantly to improving HER activity.

Additive manufacturing, popularly known as 3D printing, has been gaining significant attention in the recent past for fabricating design-controlled electrodes for electrochemical applications. Several 3D printing methods, including selective laser sintering (SLS), fused deposition modeling (FDM), and digital light processing (DLP), have been successfully used for HER electrode fabrication [2,11,12]. SLS enables the fabrication of 3D metal electrodes, whereas FDM and DLP can produce polymeric electrodes filled with electroactive materials. However, metal 3D printing is highly expensive and time-consuming due to the need for extremely long post-processing steps. On the other hand, the use of electrically non-conductive polymeric hosts in FDM and DLP produces sub-optimal performances. As a solution to this, metallization through electrochemical deposition of metals on a 3D printed construct has been investigated by several researchers [2,8,13]. Yet, these solutions are limited by low surface-to-volume ratio due to the resolution restrictions of the 3D printing methodologies involved.

To enhance the effective surface-to-volume ratio of the 3D HER electrodes, we here introduce a combination of 3D printing, pyrolysis, and electrochemical deposition. Pyrolytic carbon (PyC), produced through resin 3D printing followed by pyrolysis, is known for its good electrical conductivity and structural stability [14,15]. Furthermore, the pyrolysis process results in a substantial geometrical shrinkage (60–90%) in PyC, significantly improving the resolution of the fabrication process compared to the resin printing process [15,16]. Utilizing the conductive nature of PyC and its high surface area, we postulate 3D PyC as an efficient substrate for the electrochemical deposition of metal. In this work, we demonstrate the coating of metal bi-layers, featuring Cu and Ni, on a 3D PyC substrate for fabricating 3D electrodes for HER. We methodically study the mechanical and electrical properties of PyC structures and the effect of metal coatings on the PyC structures. These composite electrodes were further investigated for water-splitting experiments. These findings can place the presented fabrication method and architecture materials as catalyst supports for more efficient and durable catalyst systems, contributing to advancements in sustainable energy technologies.

## 2. Experimental method

### 2.1. Fabrication of 3D architected pyrolytic carbon structures

**Figure 1.**
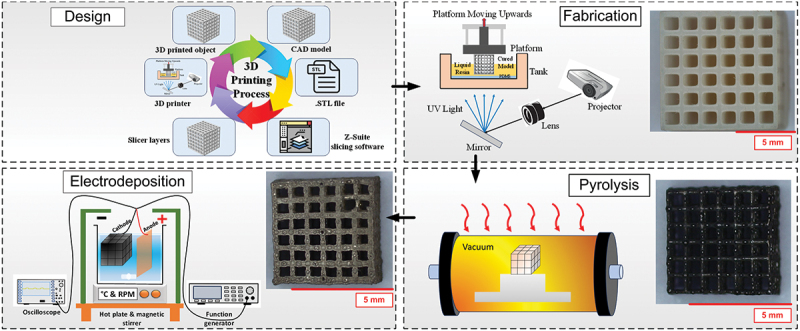
Schematic of steps involved in the fabrication of 3D structures of metallic/PyC layered materials.

Figure 1 illustrates a schematic workflow of our experimental method. Firstly, we employed SolidWorks, a computer-aided design (CAD) software by Dassault Systems, to design 3D geometries with a cubic unit cell as the prototype design. The design lattice thickness of the structures was kept constant at 400 µm, while the gap between adjacent lattices was varied to 2.8 mm, 1.9 mm, 1.5 mm, and 1.2 mm, to have four varieties of pore dimensions named Type A, Type B, Type C, and Type D, each with an overall size of 10 mm × 10 mm × 10 mm. On a planar face, the lattice geometry of A, B, C, and D featured a pore configuration of 3 × 3, 4 × 4, 5 × 5, and 6 × 6, respectively, as shown in Supplementary information (Fig. S1). Once the designs were finalized, we converted them into .stl (standard tessellation language or stereolithography) file format, which were subsequently sliced using Chitubox slicing software (CBD Technology Ltd., China) for preparing them for 3D printing with a layer thickness of 10 µm. The cubic lattice structures were additively manufactured using a Phrozen Sonic Mini 4K DLP 3D printer. The resin used for the printing was Phrozen Aqua 4K Ivory, a commercial resin from the 3D printer manufacturer. Once the printing was finished, the printed structures were washed in an isopropanol immersion for 10 minutes to remove excess unpolymerized resin, followed by a one-hour curing process in a UV chamber with a wavelength of 405 nm and an intensity of 48 watts for curing the 3D print resin to achieve the desired structures.

The 3D printed epoxy structures were carbonized in a horizontal tube furnace (Nabertherm, Germany). The heat treatment protocol was detailed in several of our previously reported articles dealing with the carbonization of polymeric precursors [17–19]. Briefly, the temperature of the furnace was increased to 350°C with a heating rate of 3°C/min, followed by an isothermal heating at 350°C for 3 h to remove any excess oxygen from the heating chamber and to allow isothermal thermochemical decomposition of the resin. Further, the temperature was raised to 900°C with a ramp of 3°C/min, followed by another isothermal heating at 900°C for 2 h. After that, the furnace was cooled down to room temperature with natural cooling. A nitrogen gas flow of 120 l/h was maintained throughout the entire process.

### 2.2. Electrochemical deposition setup

Figure 1 depicts an in-house pulse electroforming system used for metallic Cu and Ni deposition on 3D PyC structures. The setup consisted of a function generator (Siglent), an oscilloscope (Rigol), a hot plate & magnetic stirrer (Cole-Parmer, 30–550°C, 0–1100 rpm), an electrolyte solution, and electrodes. For Cu electroplating, we used copper sulfate pentahydrate (CuSO_4_.5 H_2_O, ≥98%, Sigma Aldrich, product number: 209198) as the copper source, whereas nickel sulfate hexahydrate (NiSO_4_·6 H_2_O, >98%, Sigma Aldrich, product number: 227676), nickel chloride hexahydrate (NiCl_2_·6 H_2_O, >98%, Sigma Aldrich, product number: 31462), and Boric acid (H_3_BO_3,_ Sigma Aldrich) were used for nickel deposition. The electrolyte solution was prepared by mixing 0.8 M CuSO_4_.5 H_2_O, and 0.5 M H_2_SO_4_, and Ni-based electrolyte solution was prepared using 300 ml DI water, 18 g NiCl_2_, 120 g NiSO_4_, 21 g H_3_BO_3_ separately under magnetic stirrer with a rpm of 400 and at a temperature of 40°C. Cu and Ni strips of 5 cm × 2 cm were used as the anode material, whereas the 3D PyC structures served as cathodes during the electrodeposition process. The operating parameters during pulsed electrodeposition were 100 hz pulse frequency, 40% duty cycle with square waveform, and the current density was 40 mA/cm^2^. The electrodeposited PyC structures are referred to as ECD 3D PyC in the rest of the manuscript.

### 2.3. Characterization

The 3D architectures were characterized for surface morphology and geometrical dimensions using field emission scanning electron microscopy (FESEM, Jeol, JSM-7610F). Energy dispersive X-ray spectroscopy (EDS, Oxford SDD) characterization was performed using an SEM equipped with a system using a 20 kV electron beam. To understand the effect of carbonization behavior, we performed thermogravimetric analysis (TGA) using an STA 6000 (Perkin Elmer) analyzer up to a temperature of 800°C in a nitrogen environment with a heating ramp of 5°C/min. The 3D PyC architectures were further evaluated for the mass loss (%), linear shrinkage (S, %), volume shrinkage (V, %), and structural density (ρ, mg/mm^3^ using Equations (1) (2) and (3) given below:

(1) $$S\left(\%\right)=\left(1-\frac{L_{a}}{L_{b}}\right)\times 100$$

(2) $$V\left(\%\right)=\left(1-\frac{V_{a}}{V_{b}}\right)\times 100$$

(3) $$\rho \left(\frac{\mathrm{mg}}{mm^{3}}\right)=\frac{m\left(\mathrm{mg}\right)}{V\left(mm^{3}\right)}$$

The Raman spectra of 3D PyC were acquired using a Renishaw in Via micro-Raman spectrometer with a laser wavelength of 532 nm (2.33 eV). To evaluate the composition of the carbonized materials, X-ray diffraction (XRD) was performed using a PANalytical Empyrean, Malvern Panalytical, United Kingdom setup with Cu-Kα radiation (λ = 1.54 Å) at room temperature.

To measure the thickness of ECD coatings, half of each sample was sliced, allowing for a complete view of the cross-section. The Bakelite injection molding technique involved inserting the sample into the mold, polishing it meticulously to create a cross-sectional view, and then examining the polished sample under an optical microscope (Leica, EZ4HD, Leica Microsystems, Germany) to analyze the deposited layer’s thickness. The mechanical properties of the 3D architectures studied here were measured through uniaxial compression tests with a flat punch using a hydraulic universal testing machine (Tinius, H50KL) with a load cell of 50 kN and a strain rate of 0.005 s^−1^. The electrical properties of PyC and ECD structures were evaluated utilizing a digital bench multimeter (Metravi, 45-TRMS, India). The contact angles were measured using the Drop Shape Analyzer, a contact angle goniometer, and its analysis software (KRÜSS DSA25B, Germany and KRÜSS ADVANCE, respectively).

### 2.4. Electrochemical measurements

The electrochemical investigation was conducted using Autolab 302N, Metrohm Potentiostat, and the Nova 2.1.5 software. All the electrochemical tests were done with a three-electrode setup with Ag/AgCl (saturated with KCl) and Hg/HgO (stored in 1 M NaOH) as a reference electrode and Pt mesh as a counter electrode. To assess the HER performance of the electrodeposited 3D PyC, we performed linear sweep voltammetry (LSV) and cyclic voltammetry (CV) at a scan rate of 50 mV/sec with iR correction. The potential range has been taken as 0 V to −1.80 V vs Ag/AgCl for LSV measurement and for CV it has been taken as −0.7 V to 0.5 V vs Ag/AgCl. The obtained plots were further converted to the Reversible Hydrogen Electrode (RHE) scale using Equation 4.

(4) $$E\left(\mathrm{RHE}\right)=E\left(\mathrm{Ag}/\mathrm{AgCl}\right)+0.1972+0.059\mathrm{pH}$$

For the electrochemical study, we used freshly prepared 1 M potassium hydroxide (KOH) solution in ultra-pure milli-Q water (conductivity 18.2 MΩ.cm) as an electrolyte. Frequency response analysis (FRA) was conducted from the frequency of 10,000 hz to 0.1 hz with AC perturbation of 5 mV to characterize the electrochemical impedance spectroscopy. For the calculation of electrochemical surface area (ECSA), CV was performed with different scan rates ranging from 100 mV/s to 250 mV/s in the non-Faradaic region. It involves determining the double-layer capacitance ($c_{\mathrm{dl}}$) using the CV technique in the non-faradaic region. This is done by using the formula: I = $c_{\mathrm{dl}}$× ν, where ν represents the sweep rate (mV/sec) and I is the current density at the particular fixed potential. The slope of the I v/s ν graph represents the double-layer capacitance of the electrode. The ECSA (Electrochemical Surface Area) is determined using the equation 5:

(5) $$\mathrm{ECSA}=\frac{c_{\mathrm{dl}}}{C_{s}}$$

where Cs (specific capacitance) is taken as 40 μF/cm^2^. This is a very standard procedure to calculate the ECSA and reported widely in the literature [20,21]

### 2.5. COMSOL simulation

The comprehensive simulation of compression loading was conducted using COMSOL Multiphysics 6.0 for the lattice architectures of Type A, Type B, Type C, and Type D. Both 3D PyC and ECD 3D PyC structures were evaluated for deformation and induced stresses under compression. Von Mises criterion was applied to the analysis of the induced stress. The Young's modulus and Poisson’s ratio of the 3D PyC lattices are taken as 20 GPa [22] and 0.37 [23], respectively. Further, Young’s modulus and Poisson’s ratio of ECD 3D PyC lattice were calculated using the mixture rule. Detailed calculations were presented in the supplementary information (corresponding fig. S3 and S4). The properties of the materials utilized for simulations are depicted in Table 1:

**Table 1.** Properties of the materials used for COMSOL simulation.

Properties	PyC	Ni@Cu coated PyC
Load (N)	100	100
Young’s modulus (GPa)	20	81.54
Poisson’s ratio	0.37	0.35

The .STL files of the 3D CAD models were imported as a 3D component domain in COMSOL software. Structural mechanics was applied as the governing physics for the compression test. Two surface boundary conditions were defined: a load condition and fixed constraints. The actual uniaxial compression, represented by loads directed downward along the y-axis at the top of the model and fixed constraints placed at the bottom, was simulated. Discretization of the 3D model was then conducted, followed by formulating calculation steps and executing computational analysis. Mesh convergence was performed to validate numerical results, discussed separately, with von Mises stress and displacement distributions selected as output parameters and visualized using color scales and minimum-maximum values.

## 3. Result and discussion

### 3.1. Characterization of PyC architecture

Pyrolysis of a precursor material involves multiple mass-reduction reactions, including dehydrogenation, polymer breakdown, and the production of gaseous byproducts, all of which contribute to considerable structural shrinkage [19,24]. Ensuring shape retention during pyrolysis is essential for the design and manufacturing of 3D PyC architecture. Inherent stress arising from structural shrinkage may result in structural deformation and fracture. The incorporation of lattice geometries and their spatial distribution facilitates minimal pathways for outgassing, maintaining the original geometry of the precursor with minimal structural deformities, despite significant shrinkage [25,26]. In our work, we also observed a significant shrinkage and mass loss of the 3D PyC structures, as dictated in Table 2, which aligns with findings from previous research on the pyrolysis of similar polymer precursors [27,28].

**Table 2.** Summary of additive manufactured polymer structures after pyrolysis.

Structure Type	Length, width, and height of overall structure before pyrolysis (mm)	Length, width, and height of overall structure after pyrolysis (mm)	Mass loss (%)	Structural density before pyrolysis (g/cm^3^)	Structural density after pyrolysis (g/cm^3^)	Volumetric Shrinkage (%)
A	10×10×10	4.62×4.72×4.67	85.22 ±0.12	0.078 ±0.001	0.0817 ±0.002	89.8±1.8
B	10×10×10	4.81×5.17×4.92	86.03 ±0.02	0.135 ±0.004	0.161 ±0.004	87.7±1.6
C	10×10×10	4.87×5.12×4.96	83.59 ±0.13	0.181 ±0.004	0.242 ±0.003	87.6±1.7
D	10×10×10	5.07×4.81×4.94	85.09 ±0.06	0.201 ±0.005	0.32 ±0.004	87.9±1.1

**Figure 2.**
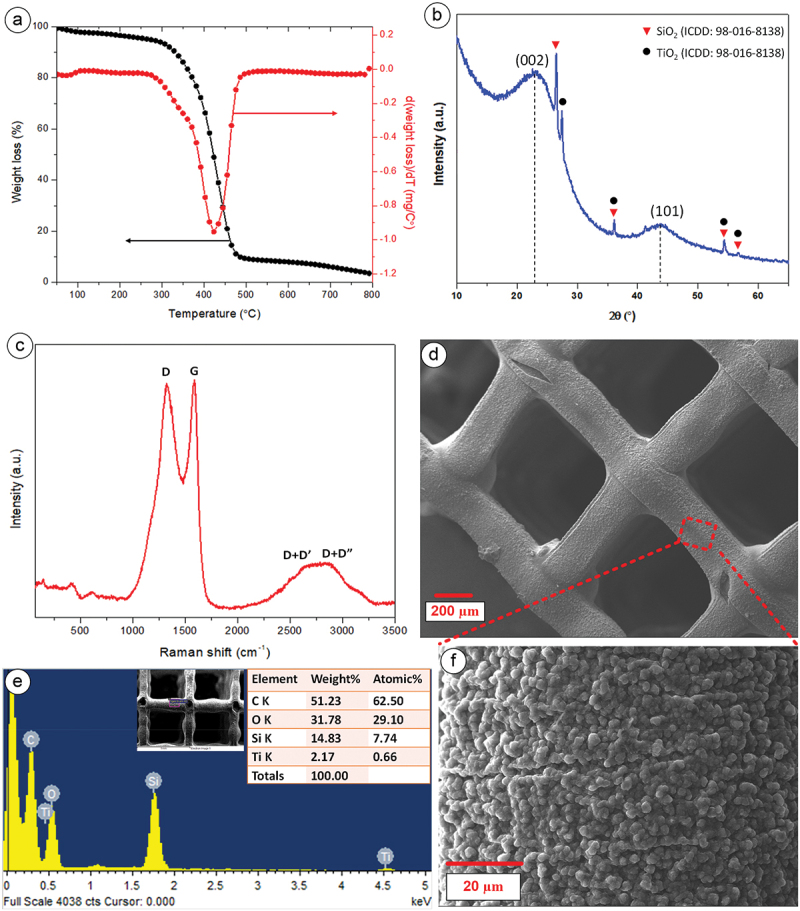
(a) TGA, (b) XRD, (c) Raman spectra of PyC structure carbonized at temperature of 900°C, (d) FESEM images of PyC microlattices, (e) elemental analysis (f) high magnification FESEM image of PyC microlattices.

The shrinkage of 3D printed resin could be correlated to weight loss with increasing temperature. To figure out the weight loss, thermogravimetric analysis (TGA) on the 3D-printed lattice was performed. Figure 2(a) presents the TGA curve of the 3D resin, depicting the mass loss with pyrolysis temperature. The resin started to degrade ($T_{\mathrm{onset}}$) at around 285°C, and the maximum weight loss ($T_{\max}$) happened at about 424°C. This weight loss was due to the resin breaking down and releasing gases. The sharp drop in weight shown by the first downward slope of the TGA curve explained why our material shrank significantly when heated to 500°C [29]. As we increased the temperature further, the material continued to lose weight. The weight loss was minimal beyond 750°C temperature. At this point, the carbon atoms in the material started to reorganize, forming graphitic layers, which could still cause some shrinkage at higher temperatures.

We performed XRD of the PyC material to characterize its composition and microstructural attributes. Distinct bulge segments within the XRD diffractogram (Figure 2(b)), positioned at approximately 2θ = 23.6° and 2θ = 44.1°, corresponded to the reflections originating from the (002) and (100) planes, revealing the insights into the degree of graphitization and the crystallinity of the carbon material. Additionally, several peaks corresponding to TiO_2_ and SiO_2_ were detected, aligning with the reference data from the ICDD (International Center for Diffraction Data) card no. 98-008-9281 and 98-016-8138, respectively. This observation could be attributed to the presence of trace amounts of Ti and Si in the resin precursor, as further confirmed by EDS.

Figure 2(c) displays the first-order Raman spectra of the 3D PyC structure, which featured two distinct peaks around 1355 cm^−1^, and 1595 cm^−1^, assigned to the D and G peaks, respectively. The D-band corresponds to the A_1 g_ mode and signifies an in-plane breathing vibration associated with the disorder within the carbon matrix, whereas the G-band corresponds to the Raman-active E_2 g_ in-plane vibration mode, indicative of the active graphitic mode of carbon [30]. The intensity ratio (I_D_/I_G_) for our 3D PyC was measured as 0.91, suggesting the presence of a substantial number of graphitic planes within a highly amorphous carbon matrix in the architected PyC, resembling a microstructure of glass-like carbon [27,28]. The peak fitting analysis of the 2D band reveals two peaks, designated as D+D’ and D+D”, at 2667 cm^−1^ and 2874 cm^−1^, respectively, which are compatible with published results [31]. It is crucial to note that the dual-peak structure found in bulk graphite’s 2D band is the result of the convolution of many peaks. However, no peaks of SiO_2_ and TiO_2_ were observed in the Raman spectra. The amount of these oxides could be minimal, and their corresponding peaks might be within the background noise of the spectra.

Figure 2(d) presents the FESEM images of the carbonized lattice structures, highlighting the unaltered geometry of the lattices. The lattice thickness of the 3D PyC was 161 ± 2 µm, whereas the average thickness of the fabricated lattices was 400 ± 2 µm (Figure S2). This translates to a linear shrinkage of ~60% for individual lattice elements. High-magnification FESEM images (Figure 2(f)) provided additional insight into the carbonized structure, revealing a granular surface texture of 3D PyC lattices, which was beneficial for anchoring metal in the subsequent electrochemical deposition, facilitating a robust interaction between the metal and PyC material. This structure resembles the formation of particle agglomerates on the surface. Additional elemental analysis utilizing EDX was conducted at five distinct locations. The average results indicated the presence of C, O, Si, and Ti, with average percentages of 52.8%, 30.7%, 14.9%, and 1.6%, respectively (see Figure 2(e)).

### 3.2. Characterization of ECD PyC architecture

**Figure 3.**
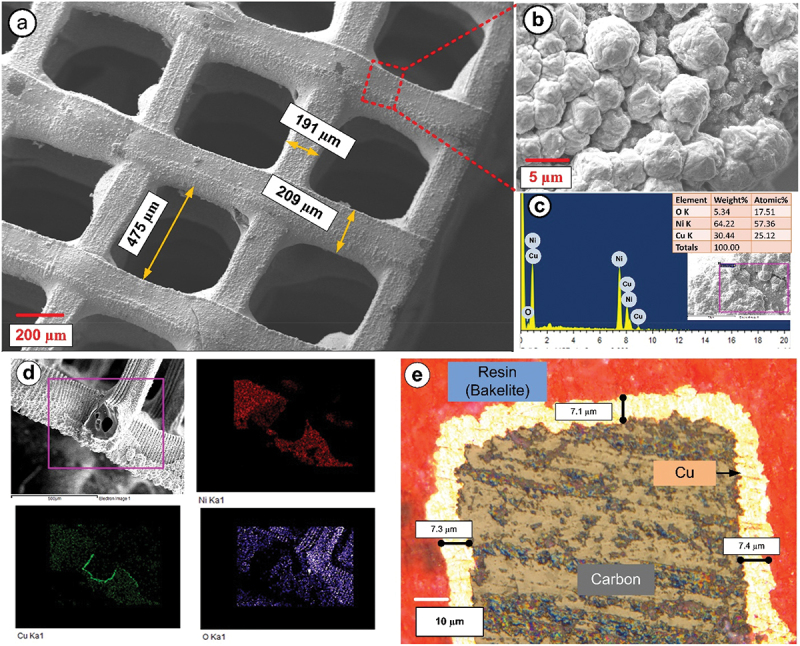
Representation of type D lattice structure (a) FESEM images and elemental analysis of Ni@Cu ECD of PyC, (b) high magnification image, (c) Ni@Cu deposited EDX results (d) area mapping and of ECD 3Dp lattice, (e) optical image of cross-sectional view of ECD 3Dp PyC.

Figure 3(a,b) illustrate the bimetallic (Ni@Cu) ECD 3D PyC Type D lattice structure. The coating exhibited a spherical granular shape, comprising a large number of irregularly smaller particles with an average size of 3.9 ± 0.1 µm. During the deposition process, the inherent electrical conductivity of the pyrolytic carbon was utilized to induce the growth of the metal phase directly onto the surfaces of the reinforcement material. To evaluate the mechanical and electrical properties, we performed four different types of metallic coatings: Ni, Cu, Ni@Cu, and Cu@Ni. The EDX spectra of a Ni@Cu-coated 3D PyC structure are presented in Figure 3(c), exhibiting the elemental composition of the sample. In this case, Cu was dep osited first to create a metallic layer over the PyC architecture, followed by Ni, resulting in a higher amount of Ni during the EDX mapping. To evaluate the bi-layer spatial distribution, the electroplated 3D sample was cut into two halves, and EDX area mapping was performed at the cross-section. The cross-sectional EDX mapping revealed the presence of a thin layer of Cu, followed by Ni on top of it (see Figure 3(d)).

The investigation into the variations in coating thickness from the interior to the exterior of the structure was conducted using optical microscopy. Interestingly, the ECD layer appeared to be thicker towards the bottom of the structure, nearer to the anode. As metal ions underwent reduction and were consumed at the cathode surface, they must be replenished by the mass transport of additional metal ions from the bulk electrolyte. However, this mass transport was hindered within the 3D cubic lattice configuration, resulting in the metal ions experiencing a lower apparent diffusion coefficient compared to the bulk electrolyte [32]. Consistency in the presence of the ECD layer was evident across all structural sizes and material combinations. The measurements obtained, as depicted in Figure 3(e), indicated an average deposition thickness of Cu approximately ~9.8 µm near the anode (see Supplementary Information fig. S3) and ~7.6 µm on the other side. Determining a specific thickness was challenging due to the intricate surface structure; however, the total average thickness of the electroplated architecture ranged between 6 and 10 µm.

**Figure 4.**
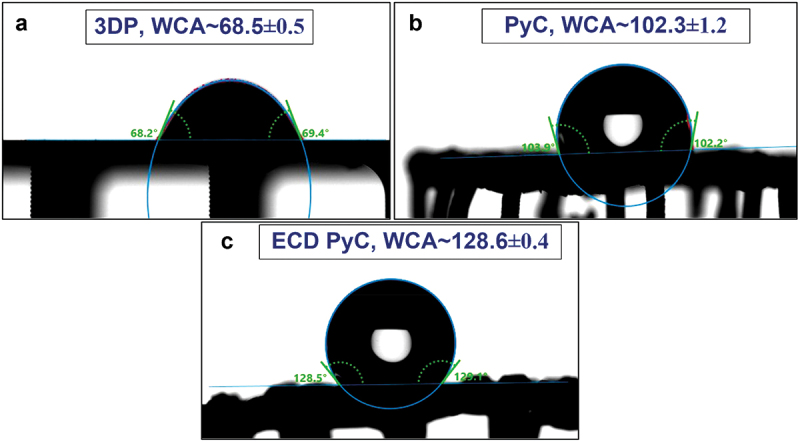
Water contact angle of type D (a) 3D resins (b) 3D PyC, and (c) Ni@Cu ECD 3D lattices.

Figure 4 displays the water contact angle (WCA) of Type D 3D resin, 3D PyC, and ECD 3D PyC architectures to evaluate their wetting behavior. Initially, we observed that the water contact angle of the 3D sample was 68.5° ± 0.5°, indicating rapid absorption or spreading of water within a few seconds. This behavior can be attributed to the relatively smooth surface with pores allowing water absorption. In contrast, the 3D PyC structures exhibited a hydrophobic nature with a WCA of 102.3° ± 1.2°. This could be attributed to the inherent hydrophobic nature of pyrolytic carbon. The surface roughness, depicted in Figure 2f, could further enhance the hydrophobicity. Metallization further increased the WCA to 128.6° ± 0.4°, which could be attributed to the rougher surface and lower surface energy observed after metallization.

### 3.3. Compressive results

**Figure 5.**
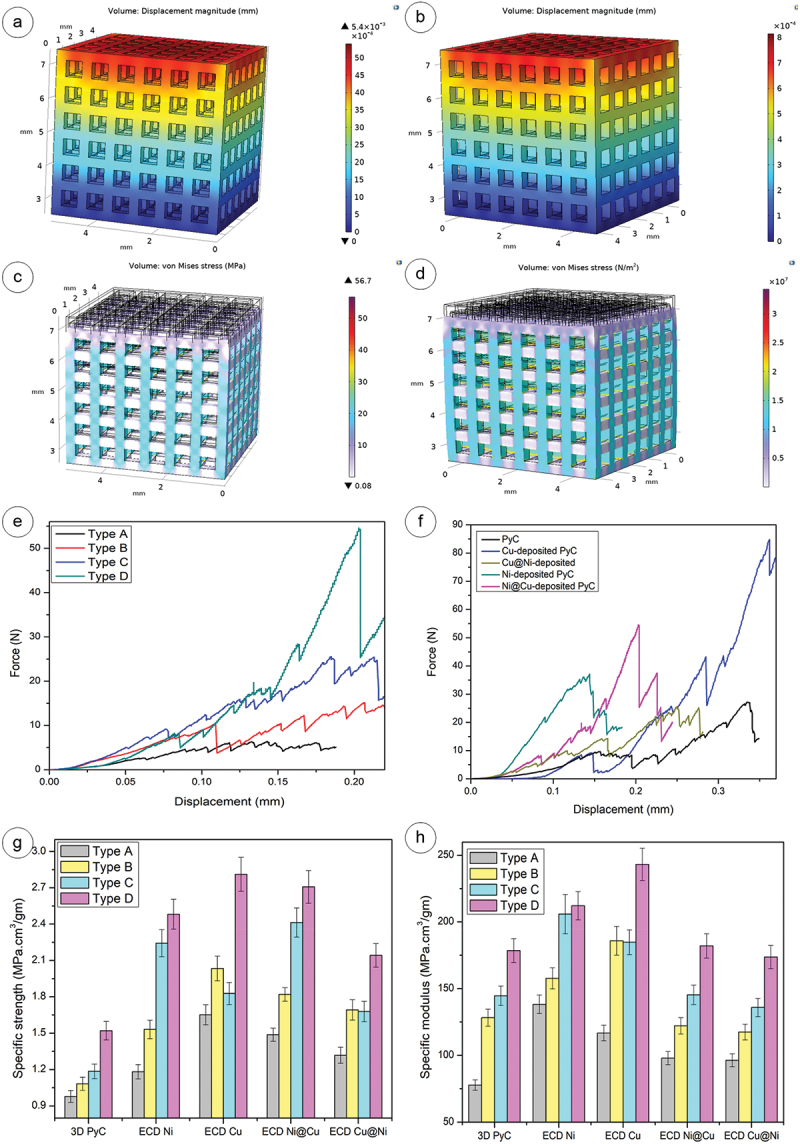
Maximum displacement of Type D structure at 100N for (a) PyC, (b) Ni@Cu ECD architecture, stress distribution of Type D structure at a load of 100N for (c) PyC and (d) Ni@Cu ECD architecture, (e) experimental load-deformation curve for ECD Ni@Cu, (f) experimental investigation on the compressive strength of Type D structure, (g) specific strength vs 3D structures, (h) specific modulus vs 3D structures.

We performed a mechanical simulation on the 3D PyC and ECD 3D PyC architectures using COMSOL Multiphysics 6.0 to see the behavior under compressive load. Figure 5(a,b) depicts the simulation results of the maximum displacement of the Type D lattice structure. As anticipated, the Type A lattice arrangements exhibited the most pronounced deformation (see Supplementary Information fig. S5) due to the larger pore sizes. In contrast, type D demonstrated exceptional stiffness and resilience, showcasing minimal displacement due to the compact nature of the lattices. For example, the average displacement of Type D lattices of ECD structures was 3, 10, and 25 times less than Type C, Type B, and Type A lattices, respectively. Moreover, the displacements in ECD structures were considerably lower compared to the 3D PyC structures due to the additional reinforcements provided by the metallic layers. For all lattice arrangements, the ECD 3D PyC exhibited around 7 times less average displacement compared to the 3D PyC structures, indicating an enhanced stiffness of the ECD 3D PyC structures, which was further confirmed by the experimental results, as presented in the later section.

Similar behavior was also observed while investigating the stress distribution within the lattice structures. The simulation results for the stress distribution are presented in Figure 5(c,d) for Type D lattices, and in Figure S6 for the other lattice types. As anticipated, Type D structures demonstrated the lowest stress levels, highlighting their superior mechanical performance and resistance to external forces. The plots of Von Mises stress vs. displacement are depicted in Figures S7, S8, S9, and S10 for Type A, Type B, Type C, and Type D, respectively. Furthermore, the stress induced in ECD 3D samples of all types was found to be 1.5 times less than that in PyC samples, attributed to the deposition of Cu and Ni enhancing its mechanical properties.

The mechanical behavior under compression loading exhibited a strong dependency on the lattice types, which was in agreement with the simulation results. Representative curves are presented in Figure 5(e) for Ni@Cu ECD structures for different lattice types. All load-deformation curves displaced a few commonalities. For example, the toe region observed at the beginning of each curve signified deformation prior to achieving complete contact between the sample and the flat punch indenter tip, a phenomenon common in cellular materials [18]. Upon complete contact with the flat punch, the lattice structures exhibited a linear region, followed by sudden drops in load in intervals, signifying brittle failures of the lattices. Such behavior was consistent with previously published reports on PyC lattices [28,33]. Furthermore, it can be observed in Figure 5(e) that structures began to break under loaded conditions, and the Type D structure was more capable of bearing load with minimal displacement compared to other types of lattices. Type D structures showed an increase in mechanical resilience with an average of 10.2, 5.5, and 2.3 times that of Type A, Type B, and Type C lattices, respectively. Mostly the lattice structure starts breaking from the toe region, and then parallel crystallographic slip offset were observed in several lattice member. On the other hand, the dependency on the materials system is demonstrated in Figure 5(f), where the load-displacement curves are plotted for different materials for Type D lattices. Figure S11 depicts the load-displacement curves for the other lattice types. For all lattice types, it could be clearly observed that metallization improved the compressive properties of the brittle PyC structures, enabling them to withstand higher loads. Among the four types of ECD structures, Cu-coated structures exhibited the maximum compressive load before failure, attributed to the ductile nature of copper, which allowed for greater deformation and energy absorption compared to other materials. The maximum compressive load before failure of the Cu-plated lattice was observed at approximately 85 N.

To compare the mechanical behavior of all the material systems, we plotted the specific strength and specific modulus in Figures 5(g,h), respectively. The absolute values of strength and modulus are presented in Figure S12. It could be clearly observed that lattice Type D exhibited the best mechanical properties among all the lattice types due to the spatial compactness. For example, for Cu-coated PyC structures, the specific strength of Type A lattices was 1.65 ± 0.1 MPa.cm^3^/g, whereas it increased to 2.82 ± 0.21 MPa.cm^3^/g for Type D. For all lattice types, metallization improved the mechanical properties significantly compared to the pristine 3D PyC materials, whereas Cu-coated PyC structures exhibited the best properties. While comparing to 3D PyC structures, the improvement in specific strength and modulus for Cu-coated PyC structures was 1.9 and 1.5 times, respectively. Ni coating resulted in the poorest performance among the metalized structures. Such behavior of Ni coating was also reported in reference [34]. In comparison to mono-metallic coating, the bimetallic structures showed intermediate properties, whereas no significant difference was observed between Cu@Ni and Ni@Cu structures.

### 3.5. Electrical demonstrator

**Figure 6.**
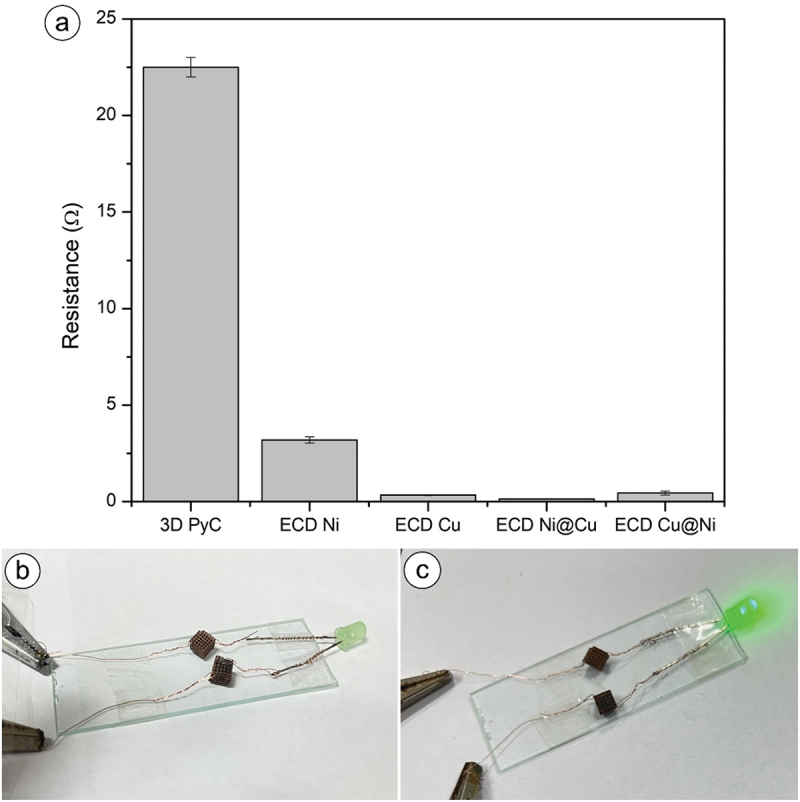
(a) Electrical resistance graph, (b) LED setup when power source was off, (c) LED setup when power source was on.

Figure 6(a) shows the electrical resistivity values of 3D PyC and ECD 3D PyC structures. Notably, the pyrolysis steps converted the electrically non-conductive resin into electrically conductive PyC even though the electrical property of PyC was significantly poorer than metals. Consequently, our PyC structure exhibited the highest resistance value among the different material systems studied here. However, the electrical property of PyC was enough to facilitate electrochemical deposition. Subsequent to the high resistance of PyC, a systematic decrement is observed in the following order: Ni>Cu@Ni>Cu>Ni@Cu. The resistivity values for polycrystalline copper and nickel in bulk form at 298 K have been reported as 1.67 × 10^−6^ ohm-cm and 6.8 × 10^−6^ ohm-cm, respectively [35]. This means that copper has a lower resistivity compared to nickel at room temperature, making it a more efficient conductor of electricity. This progression highlighted the impact of electrodeposition, particularly with the introduction of Ni and Cu composite layers, on improving the electrical conductivity of the samples. To further demonstrate the excellent electrical properties of the layered structures, we incorporated two identical ECD structures in an electrical circuit for an LED bulb. Figures 6(b,c) depict the LED connection and its operational states, both when the current was off and on, respectively, signifying the compatibility of ECD structures as electrical components.

### 3.6. Electrochemical results

**Figure 7.**
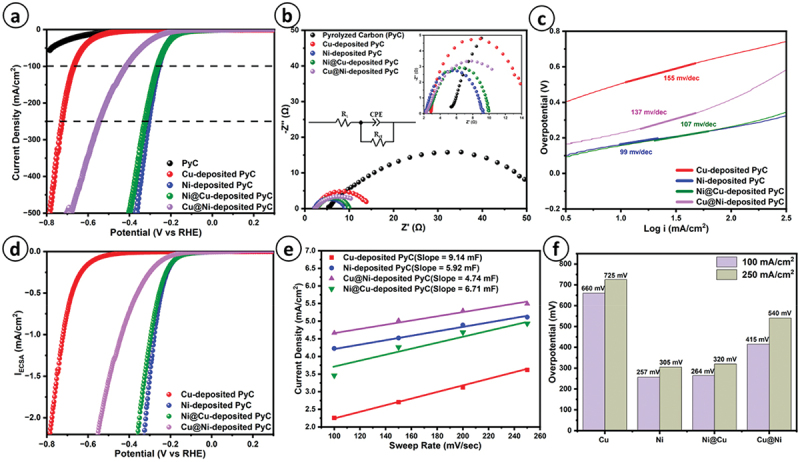
(a) iR corrected polarization curve, (b) electrochemical impedance spectroscopy (inset of fig. provides the circuit fitting and resistance to charge transfer), (c) tafe plot, (d) ECSA based polarization curve, (e) double layer capacitance plot, (f) overpotential required to achieving 100 mA/cm^2^ and 250 mA/cm^2^ for different materials.

Electrode material and geometry have a significant effect on the HER overpotential as well as onset. We performed experiments on four different lattice structures and found the best suitable results with Type D architecture. Further, in this section, a comparative analysis of Type D ECD microlattices was conducted based on the overpotential necessary to achieve a current density of 100 mA/cm^2^ and 250 mA/cm^2^. On the basis of the polarization curve shown in Figure 7(a), it is evident that among all the catalysts, the Ni-coated PyC exhibited remarkable activity as a HER electrocatalyst. The heightened HER activity of ECD Ni may be attributed to its improved electronic conductivity, enhanced redox chemistry, and quicker HER kinetics. This catalyst only needed an overpotential of 257 mV to obtain a current density of 100 mA/cm^2^ and 305 mV for a current density of 250 mA/cm^2^. We conducted electrochemical impedance spectroscopy in the potential region beyond onset to determine the charge transport behavior. The Z-view software was used to perform an equivalent circuit fitting. The fitted circuit, shown in the inset of Figure 7 (b), consisted of a resistance in series, known as the solution resistance (R_s_), and parallel components, including a constant phase element (CPE) and a charge transfer resistance (R_c_). When comparing electrodeposited metals to pristine PyC, both the resistance to charge transfer and the resistance to the solution were significantly reduced, which indicated that metallic electrodeposition provided a less resistive path for the adsorption of ions and release of H_2_. Based on the Nyquist Plot shown in Figure 7(b), the exceptional activity of Ni-deposited PyC for the HER may be attributed to the efficient and rapid transfer of charge to the exposed active sites of Ni. The charge transfer resistance of PyC with Ni deposition was significantly reduced to 4.75 Ω, in contrast to the pristine PyC, which exhibited a charge transfer resistance of 52.4 Ω. The impressive performance of Ni-deposited PyC could be attributed to the rapid HER kinetics over the Ni surface, as indicated by the Tafel analysis in Figure 7(c), which reveals a minimum Tafel slope of 99 mV/dec and a maximum of 155 mV/dec for Cu in comparison to Ni@Cu (106 mV/dec) and Cu@Ni (137 mV/dec).

**Figure 8.**
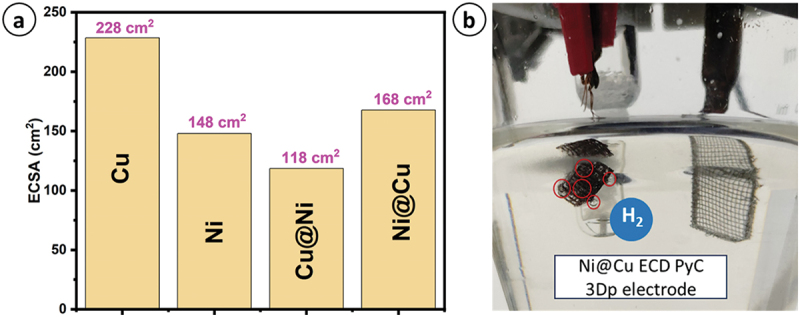
(a) Plot for electrochemical active surface area for different materials, (b) bubbling on Ni@Cu ECD 3D electrode for HER application.

In order to determine the electrochemical active surface area (ECSA), as seen in Figure 8 (a), we needed to conduct CV at various scan rates to get the double-layer capacitance (C_dl_) which is shown in Figure 7 (e). The specific capacitance was taken as 40 µF-cm^−2^ in this case due to the alkaline electrolyte involved [20]. A greater HER activity might arise from either an increased ECSA or a higher conversion rate per unit of active sites, which is referred to as a specific activity. To further investigate the HER specific activity, we assessed the electrochemically active surface area based current density (specific activity) shown in Figure 7(d). Ni-deposited PyC material had the greatest specific activity and surpasses other materials, as shown in Figure 7(d). This indicates that it needed fewer active sites to produce hydrogen. The comparative analysis of other catalysts’ activity is presented in Figure 7(f), which showed that incorporating Ni metals resulted in excellent HER activity. The bare PyC exhibited less activity owing to its low conductivity and reduced reactivity. The onset potentials of each electrode exhibited notable variations. For example, pristine PyC had an onset potential of −560 mV, whereas Cu-, and Cu@Ni-deposited PyC had an onset potential of −480 mV and −254 mV, respectively. On the other hand, Ni- and Ni@Cu-coated PyC electrodes had almost the same onset potential of just −180 mV. The three-electrode setup as illustrated in Figure 8(b), a substantial amount of bubbling occurred as hydrogen is formed at the ECD 3D PyC lattices and oxygen is formed at the Pt mesh.

**Figure 9.**
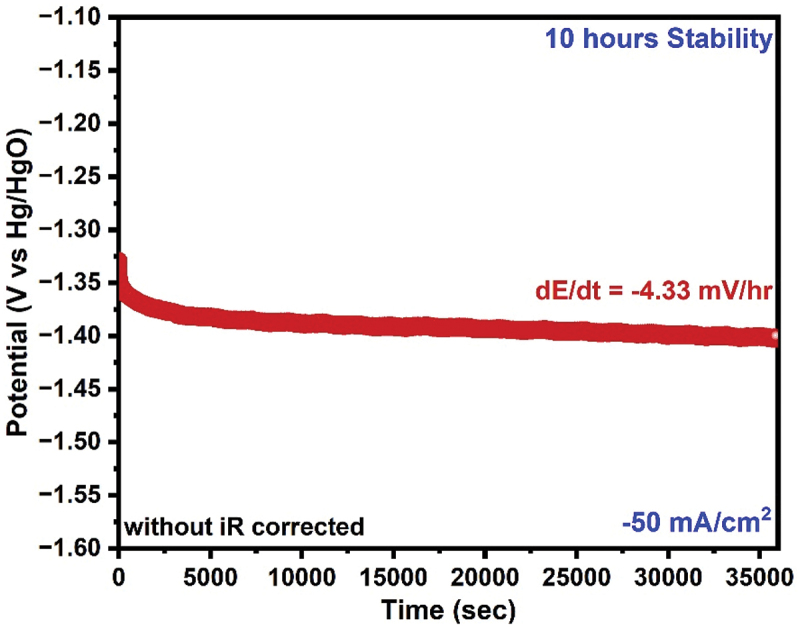
HER stability test at −50 mA/Cm^2^ for Ni@Cu PyC.

The result of Ni@Cu was considered the best for HER application evaluating various mechanical and electrical characteristics because the Cu layer (thickness: 8 ± 0.6 μm) penetrated the holes and pores of the rough PyC substrate, the Ni layer exhibited a continuous, packed layer with a thickness of 10 ± 1 μm. Alongside a comprehensive electrochemical investigation of our samples, it is crucial to evaluate their stability for prolonged HER application. To check the stability test that employs Hg/HgO as a reference electrode for our optimally deposited Ni@Cu PyC electrode. Our electrode exhibits exceptional stability during a 10-hour operation at a high applied current of −50 mA/cm^2^. Our electrodeposited material exhibits an exceptionally low potential increment of −4.33 mV/h, as illustrated in Figure 9.

The comparative data in Table 3 shows the overpotential required to achieve specific current densities in an alkaline medium, along with the corresponding Tafel slopes for the present results. As shown, many researchers have explored additive manufacturing for fabricating 3D electrocatalysts for HER, often focusing on metal 3D printing or metallic deposition methods. In contrast, we employed a novel approach by combining 3D printing, pyrolysis, and electrodeposition to produce a 3D electrocatalyst. The Ni@Cu-coated PyC from this study exhibits competitive electrochemical performance for HER, with a Tafel slope of 107 mV/dec and an overpotential of 264 mV in 1 M KOH at 100 mA/cm^2^. Although materials like Ni-Pt and Ni-Co show slightly better Tafel slopes, our method offers a more cost-effective alternative by using 3D printing and electrodeposition, avoiding the need for expensive noble metals like platinum. Furthermore, the bimetallic Ni@Cu coating ensures uniformity and enhances both mechanical and electrochemical performance, making it highly suitable for practical applications.

**Table 3.** Comparison of HER activity of ECD 3D PyC electrode catalysts with other 3D printed electrodes in an alkaline medium.

3D printed active electrode materials	Electrolyte	Current density(mA/cm^2^)	Tafel slope (mV/dec)	Overpotential (mV)	Reference
NiMo	1 M KOH	10	NA	45	[36]
Graphene Ni-Co	1 M KOH	10	164.65	101.92	[8]
Graphene Ni-Pt	1 M KOH	10	139.1	268	[37]
Mo-Ni	0.1 M KOH	1500	NA	325	[38]
Graphene NI-Fe-P	1 M KOH	100	162	341	[39]
Graphene CoNiCH	1 M NaOH	100	74.8	322	[40]
Ni-Pt	30 wt% KOH	10	NA	152.2	[41]
Ni@Cu-coated PyC	1 M KOH	100	107	264	Present work

## 4. Conclusion

To summarize, we presented the electrodeposited metallic bi-layer PyC electrode as a cost-effective electrode for HER applications. The electrodes were fabricated using resin 3D printing, followed by carbonization, and electrochemical deposition of Cu and Ni on the 3D PyC substrate. The carbonization process yielded significant geometrical shrinkage (~60% shrinkage in lattice thickness and ~88% volumetric shrinkage) into the 3D printed shape, enhancing the geometric resolution and effective surface-to-volume ratio. The electrodeposition step led to a metal thickness of 6–10 µm on the PyC surface, which further enhanced the mechanical and electrical properties of 3D PyC structures. The Cu-coated PyC structures exhibited the highest mechanical properties, with a specific strength of 2.8 ± 0.15 MPa.cm^3^/g and a specific modulus of 243 ± 12 MPa.cm^3^/g. In contrast, Ni-coated PyC turned out to be the mechanically poorest among the metalized materials. The bi-metallic (Cu@Ni and Ni@Cu) 3D PyC structures exhibited intermediate mechanical properties, demonstrating the coherence attributes of both metallic components under compressive loading. Upon investigating their utility in HER experiments, Ni@Cu-coated PyC materials resulted in the lowest HER overpotential by achieving a low HER overpotential of 264 mV (100 mA/cm^2^, KOH (1 M, aq)) with a Tafel slope of 107 mV/dec^1^, demonstrating it the best choice for HER electrode material among the studied samples here. Our work can potentially open up a new avenue for HER electrode fabrication. Future research in this area should explore variations and control over geometry, as well as further deposition of combinations of transition metal-based coatings. Even though the current work only focused on cubic lattices, other lattice types could be employed to enhance the material properties of the metamaterial electrode. Furthermore, this proof-of-concept study did not reach the fabrication resolution, as the focus was only to demonstrate the fabrication methodology.

## Supplementary Material

Supplemental Material
