# Supplementary material for: Bi-metallic electrochemical deposition on 3D pyrolytic carbon architectures for potential application in hydrogen evolution reaction
Source: Sci Technol Adv Mater. 2024 Oct 29;25(1):2421740. doi: 10.1080/14686996.2024.2421740 (PMC11544737; doi:10.1080/14686996.2024.2421740)
Supplement: Supplemental Material [file TSTA_A_2421740_SM4127.docx]

**Bi-metallic Electrochemical Deposition on 3D Pyrolytic Carbon Architectures for Potential Application in Hydrogen Evolution Reaction**

Prince Kumar Rai^1^, Amritanshu Singh^1^, Shashwat Bishwanathan^2^, Prashant Kumar Gupta^2^, De Yi Wang^3^, Monsur Islam^3,*^, Ankur Gupta^1,*^

^1^Department of Mechanical Engineering, Indian Institute of Technology Jodhpur 342030, India.

^2^Department of Chemical Engineering, Indian Institute of Technology Jodhpur 342030, India.

^3^IMDEA Materials Institute, Calle Eric Kandel, 2, 28906 Getafe, Madrid, Spain.

*Corresponding author: [ankurgupta@iitj.ac.in](mailto:ankurgupta@iitj.ac.in) (A.G.); [monsurislam79@gmail.com](mailto:monsurislam79@gmail.com) (M.I.)

**Supplementary Document**

On a planar face, the lattice geometry of A, B, C, and D featured a pore configuration of 3×3, 4×4, 5×5, and 6×6, respectively, as shown in Fig. S1.

**
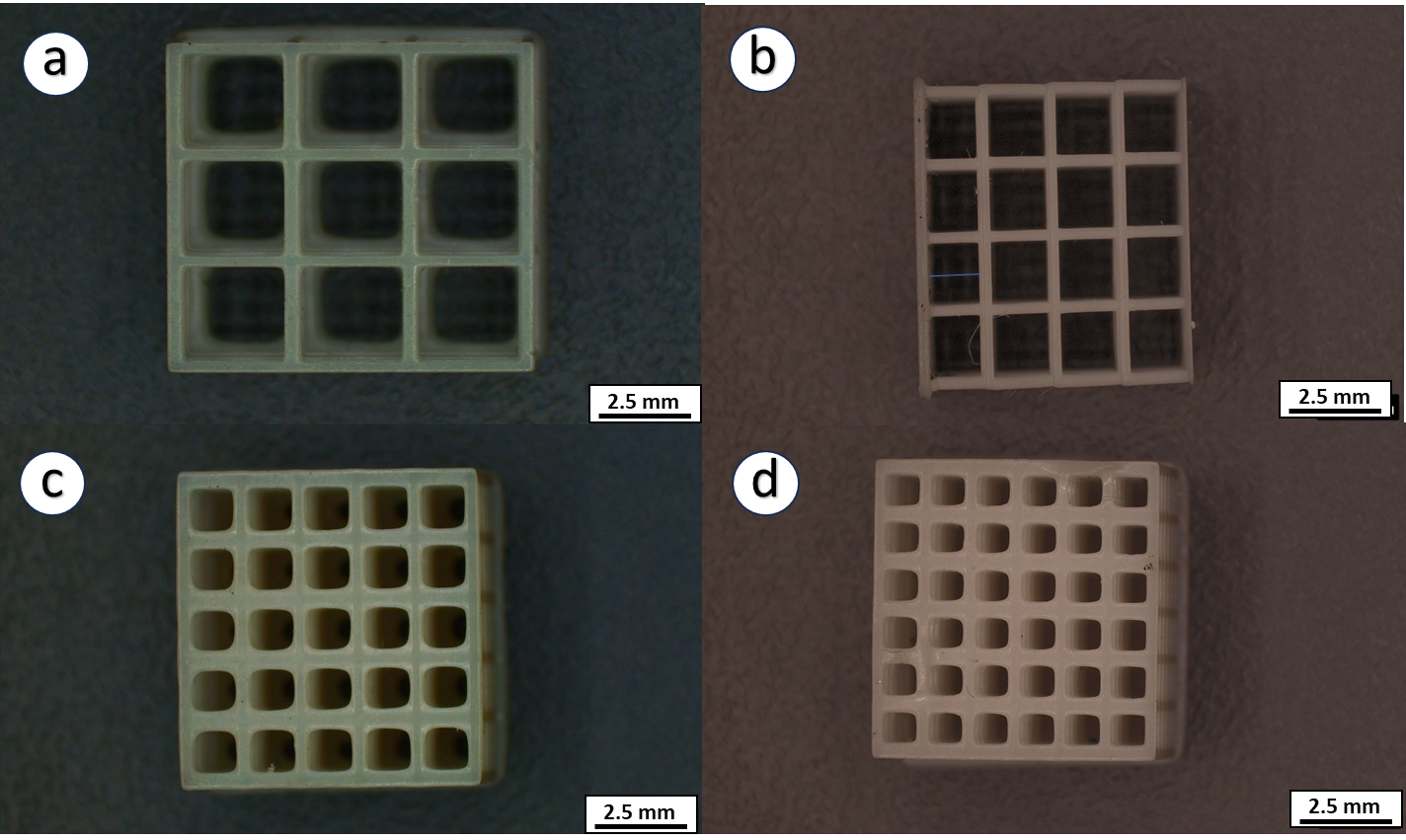
**

Figure S1. Macroscopic images of the lattice geometry of (a) A, (b) B, (c) C, and (d) D featured a pore configuration of 3×3, 4×4, 5×5, and 6×6, respectively

Figure S2. FESEM images of 3D printed cubic mesh structure of (a) type A (b) type D

Figure S2 depicts FESEM images of 3DP lattice structures before pyrolysis. We successfully constructed a consistent square pattern of horizontal and vertical lattices with an average thickness of 400±5 μm using DLP 3D printing, as shown in Figure S2 (a). FESEM images were captured for the 3D printed samples of type A and type D providing a detailed visual analysis of their structural characteristics, with varying distances between adjacent lattices. Higher magnification FESEM images (inset of Figure S2 (a)) show a smooth surface texture for the 3D resin structures. Additionally, precise measurements of dimensions were conducted, offering quantitative insights into the morphological features of the samples.

The measurements obtained, as depicted in Figure S3, indicate an average deposition thickness of approximately ~9.8 µm near the anode and ~7.6 µm on the other side.

Figure S3. Optical images of cross-section of Cu deposited on 3Dp architecture

The Young’s modulus and Poisson’s ratio of the EDC 3Dp PyC lattice can be calculated using the mixture rule. The Rule of Mixtures for Young's modulus states that the effective modulus of a composite material is calculated by taking a weighted average of the moduli of its parts, with the weights determined by their volume fractions i.e. $E_{eff}=\sum_{i=1}^{n} V_{i}E_{i}$ , where V is the volume fraction. Similarly, the effective poisons ratio can be calculated as, $\mu_{eff}=\sum_{i=1}^{n} V_{i}\mu_{i}$.

The below image shows the cross-sectional view of the lattice of the limb for the unit width. The center layer is the PyC carbon surrounded by ECD copper and nickel layer of average thickness 8 microns respectively.


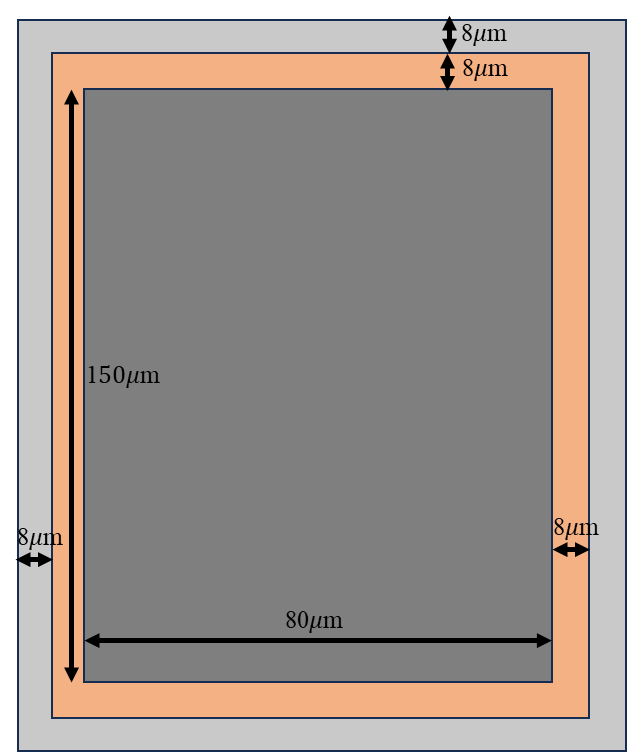


Figure S4. Schematic of one limb coated with Cu and Ni layer

Now, the volume of the fraction of individual elements can be calculated as follows:

$V_{c}=\frac{12000}{20384}=0.588$, $V_{Cu}=\frac{3936}{20384}=0.193$ and $V_{Ni}=\frac{4448}{20384}=0.218$

Now, the effective Young’s modulus can be calculated as:

$$E_{eff}=\sum_{i=1}^{n} V_{i}E_{i}$$

$$E_{eff}=0.588\times20+0.193\times130+0.218\times205$$

$$E_{eff}=81.54GPa$$

Similarly, the effective Poisson ratio can be calculated as:

$$\mu_{eff}=\sum_{i=1}^{n} V_{i}\mu_{i}$$

$$\mu_{eff}=0.588\times0.37+0.193\times0.31+0.218\times0.35$$

$$\mu_{eff}=0.35$$

Figure S5 depicts the simulation results of the maximum displacement of the type A, type B, and type C lattice structures. The type A lattice arrangements exhibited the most pronounced deformation.

Figure S5. Maximum displacement at 100N for type A, type B, and type C structures

Figure S6. Stress distribution at 100N for type A, type B, and type C structures

The below figure depicts the variation of Von Mises stress with the vertical displacements. It can be observed from the below graphs that the maximum stress is induced in as received 3Dp lattice whereas the minimum stress is induced in the case of ECD 3Dp PyC. Further, comparing ECD 3Dp samples of type B with 3Dp PyC, the stress induced in ECD 3Dp samples was found to be less (1.5 times) than that in 3Dp PyC samples, attributed to the deposition of Cu and Ni enhancing its mechanical properties. Also, type A exhibited the highest stress values across the mesh, indicating greater susceptibility to mechanical stresses and potential weak areas or structural inefficiencies.

Figure S7. Von Mises Stress vs displacement, and Von Mises Stress vs lattice thickness (inset) plot for Type A at a load of 100N for (a) 3D PyC, and (b) 3D ECD architecture of type A using COMSOL Multiphysics 6.0

For type B lattices, comparing ECD 3Dp samples with 3Dp PyC, it can be concluded that the stress induced in ECD 3Dp samples was found to be less than that in 3Dp PyC samples. Also, the stress-induced in type B lattices is less as compared to type A due to their structural efficiencies.


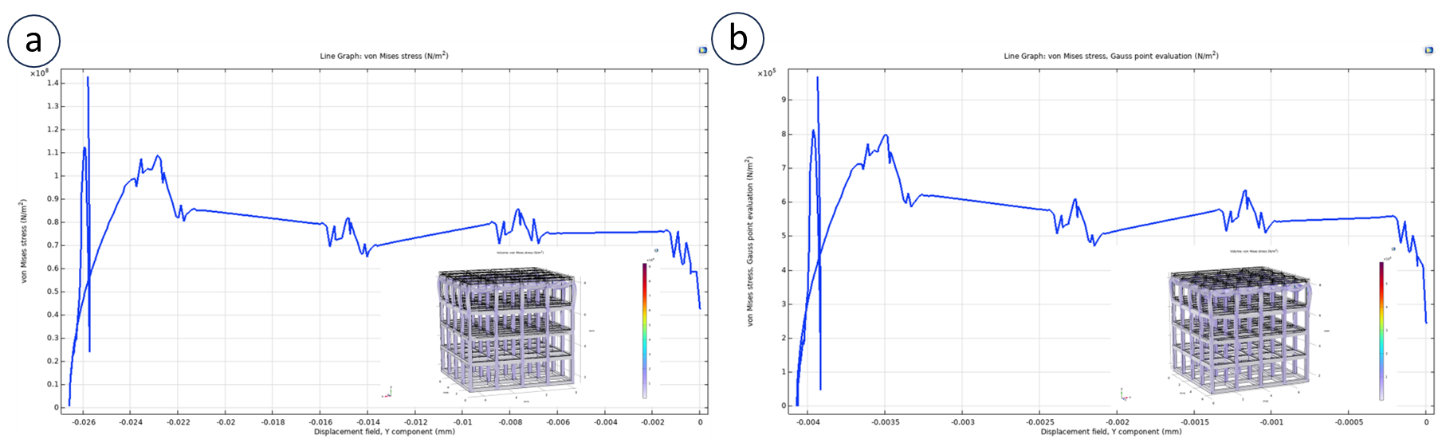


Figure S8. Von Mises Stress vs displacement, and Von Mises Stress vs lattice thickness (inset) plot for Type B at a load of 100N for (a) 3D PyC, and (b) 3D ECD architecture of type B using COMSOL Multiphysics 6.0

Similarly, for type B lattices, comparing ECD 3Dp samples with 3Dp PyC, it can be concluded that the stress-induced in ECD 3Dp samples was found to be less than that in 3Dp PyC samples. Also, the stress-induced in type C lattices is less as compared to type A and B due to their structural efficiencies.


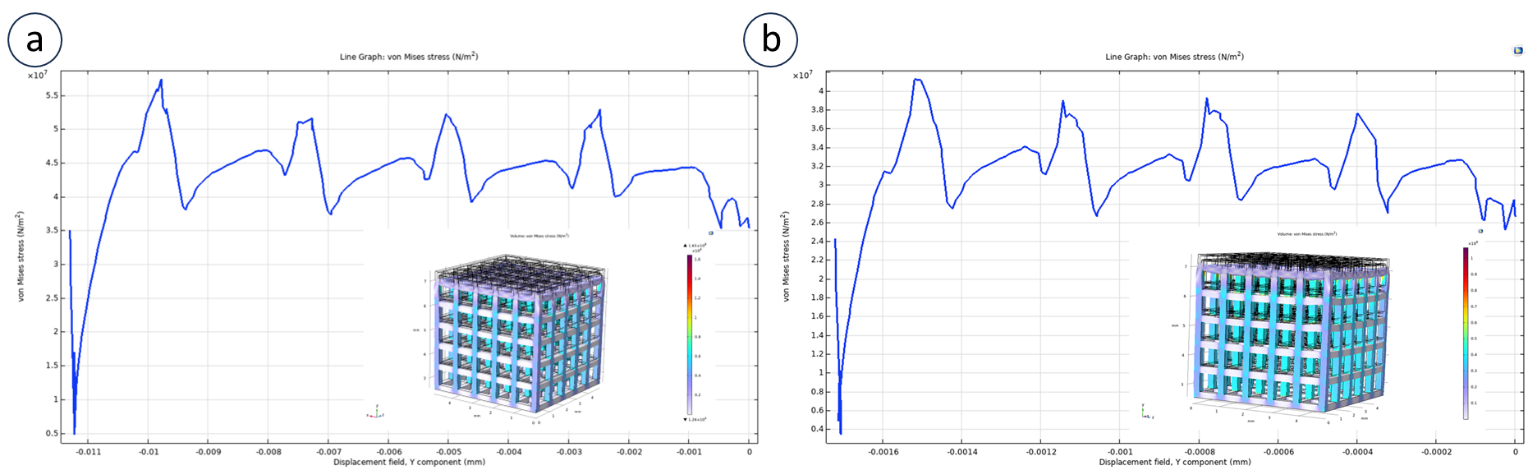


Figure S9. Von Mises Stress vs displacement, and Von Mises Stress vs lattice thickness (inset) plot for Type C at a load of 100N for (a) 3D PyC, and (b) 3D ECD architecture of type C using COMSOL Multiphysics 6.0


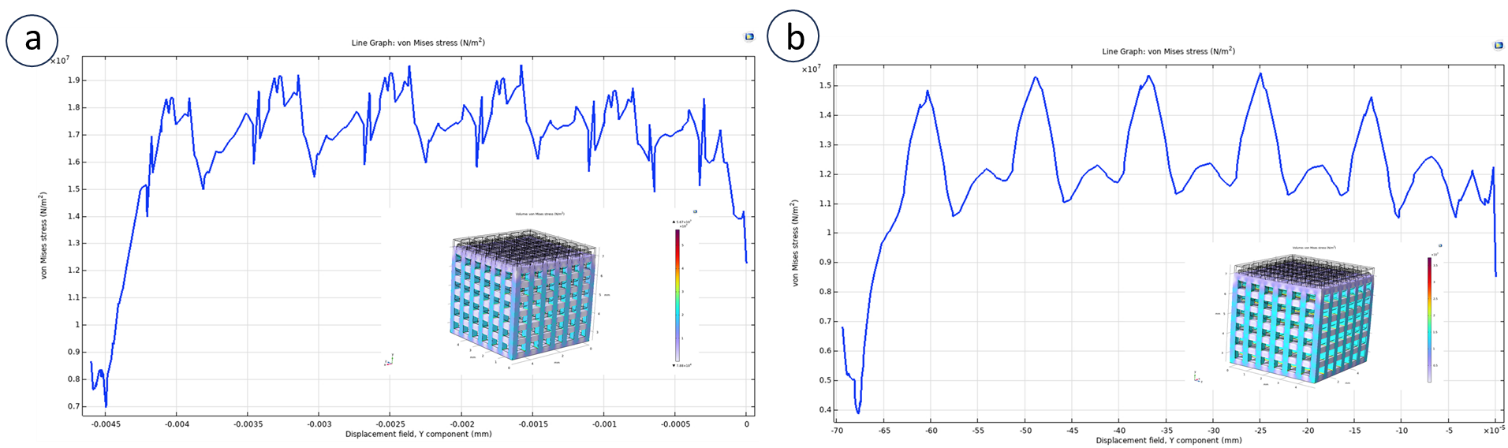


Figure S10. Von Mises Stress vs displacement, and Von Mises Stress vs lattice thickness (inset) plot for Type D at a load of 100N for (a) 3D PyC, and (b) 3D ECD architecture of type C using COMSOL Multiphysics 6.0

Figure S11. Experimental investigation on the compressive strength of (a) Type A, (b) Type B, (C) Type C structure

The compression modulus was determined by calculating the slope of the initial linear section of the stress-strain curve. Figure S12 (a) demonstrates that the resin lattice of type D exhibits the highest compressive strength (1.5MPa), whereas the 3D PyC lattice of type A shows the lowest compressive strength (0.5 MPa). The compression strength was defined as the highest value of the first peak in the stress-strain curves. Similarly, Figure S12 (b) shows that the compression modulus, which is the slope of the stress-strain curve, is highest (75 MPa) for the ECD 3D PyC lattice structure of type D and lowest (5MPa) for the resin structure of type A.


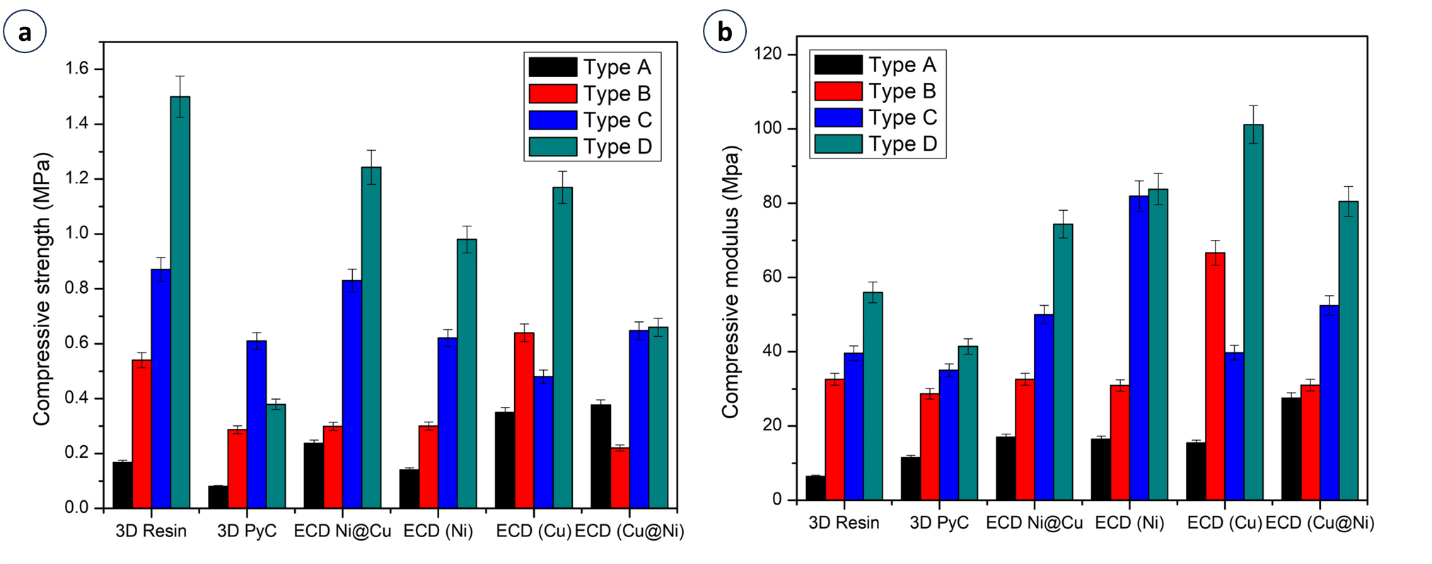


Figure S12. (a) Compressive strength vs 3D structures, (b) Compressive modulus vs 3D structures

Figure S13. Setup utilized for resistance calculation
